# Optimization of Phenolic Compounds Extraction and Antioxidant Activity from *Inonotus hispidus* Using Ultrasound-Assisted Extraction Technology

**DOI:** 10.3390/metabo13040524

**Published:** 2023-04-05

**Authors:** Liliana Machado-Carvalho, Tânia Martins, Alfredo Aires, Guilhermina Marques

**Affiliations:** 1 Department of Agronomy, School of Agrarian and Veterinary Sciences, University of Trás-os-Montes and Alto Douro (UTAD), 5000-801 Vila Real, Portugal; lilianac@utad.pt (L.M.-C.); taniam@utad.pt (T.M.); alfredoa@utad.pt (A.A.); 2 CITAB—Centre for the Research and Technology of Agro-Environment and Biological Sciences, UTAD, 5000-801 Vila Real, Portugal; 3 Inov4Agro—Institute for Innovation, Capacity Building and Sustainability of Agri-Food Production, UTAD, 5000-801 Vila Real, Portugal

**Keywords:** antiradical activity, *Inonotus hispidus*, polyphenol content, response surface methodology, ultrasound-assisted extraction

## Abstract

The use of ultrasound-assisted extraction (UAE) of bioactive compounds has been increasing because it is a good alternative to the conventional extraction methods. UAE was used to maximize total polyphenol content (TPC), 2,2-diphenyl-1-picrylhydrazyl (DPPH) scavenging capacity, and ferric reducing antioxidant power (FRAP) of the mushroom *Inonotus hispidus* using response surface methodology (RSM). Firstly, the effect of 40% (*v*/*v*) ethanol and 80% (*v*/*v*) methanol on the TPC, DPPH scavenging capacity, and FRAP was evaluated. The ethanolic extracts showed a significantly higher (*p* < 0.0001) TPC, DPPH scavenging capacity, and FRAP than the methanolic extracts. The best condition to produce an extract with the higher TPC and antioxidant activity was achieved when using 40% (*v*/*v*) ethanol, a ratio of 75 mL/g, and an extraction time of 20 min. The chromatographic profile of the extract obtained in the optimized condition revealed that hispidin is the main polyphenol present in the extracts of *I. hispidus*, representing, together with hispidin-like compounds, the majority of the phenolic compounds (159.56 µg/g DW out of 219.01 µg/g DW). The model allowed us to optimize the conditions to maximize the extraction of phenolic compounds with antioxidant activity from *I. hispidus*, demonstrating its potential as a source of antioxidant compounds, with possible industrial, pharmaceutical, and food applications.

## 1. Introduction

The medicinal properties of mushrooms have been the focus of recent reviews [1,2,3,4]. Their health benefits are as diverse as anti-inflammatory, antioxidant, antiviral, antibacterial, antifungal, anticarcinogenic, antidiabetic, and hypoglycemic, as well as their cosmeceutical potential, among others [1,2,3,4,5,6,7,8]. *Inonotus hispidus* (Bull.) P. Karst. (Hymenochaetaceae), commonly known as shaggy bracket, is a plant pathogen fungus, particularly of deciduous trees such as *Fraxinus*, *Quercus*, *Sorbus*, and *Malus* [9]. It is a medicinal mushroom with various health benefits such as anticancer and immunomodulatory activities, among others [10,11,12]. Over the last few decades, several studies have demonstrated the antiviral [13], anti-tumor [11,14], antioxidant [15,16,17], antifungal [18], antiobesity [12], and hepatoprotective effects [19] of this mushroom. The beneficial biological effects of mushrooms are usually attributed to polysaccharides and phenolic compounds that exhibit a great free radical and reactive oxygen species scavenging power [20,21]. The extraction of such bioactive compounds largely depends on the effectiveness and efficiency of the selected extraction methods [22]. Thus, the use of an extraction method capable of maximizing the extraction of those compounds from mushrooms, combined with a green, fast, and economical method, is of great importance.

Conventional methodologies such as maceration and Soxhlet are generally simple and easy to perform and are based on the nature of the solvent and external factors such as temperature, time, and agitation, which help to increase the solubility of compounds in that solvent [23]. Nevertheless, such conventional techniques generally involve long extraction periods, require high volumes of solvent, and result in low extraction yields [24]. Ultrasound–Assisted Extraction (UAE) is a technique that has been used in the last years to replace other conventional extraction methods. The reduction in extraction time, energy, and the use of solvents are some of the advantages of UAE. High-power ultrasound can be applied using two types of devices, ultrasonic bath, or probe-type ultrasound equipment [25]. However, the probe system is more powerful due to an ultrasonic intensity delivered through a smaller surface (the tip of the probe), when compared to the ultrasonic bath. The immersion of the probe into the reactor also leads to the direct delivery of ultrasound in the extraction solution, resulting in minimal ultrasonic energy loss [25]. When using UAE, bubbles are formed by the expansion and compression cycles generated by ultrasound waves [22,25]. The collapse of these bubbles generates a localized pressure that disrupts the cell walls in the matrix, improving the release of intracellular substances into the solvent [22,25], thus resulting in a higher extraction yield with a shorter extraction time. Despite this, the extraction process yield is still affected by other variables such as the solvent-to-solid ratio, the type of solvent and its concentration, time of contact, and temperature [26,27,28]. Therefore, for each raw material, it is important to define the conditions that maximize the recovery of the compounds of interest. 

To our knowledge, there are no studies in the literature on the extraction of phenolic compounds from *I. hispidus*. Accordingly, the purpose of this study was to select the best extraction conditions using Response Surface Methodology (RSM) regarding the solvent-to-solid ratio and time of contact using UAE to maximize the recovery of antioxidant phenolic compounds from *I. hispidus* and to profile the extract obtained in the optimized conditions by High-Performance Liquid Chromatography with a Diode Array Detector (HPLC-DAD). Firstly, the efficiency of two solvents (ethanol and methanol) in the extraction of phenolic compounds was evaluated. Subsequently, UAE was performed using the selected solvent, and the effects of different solvent-to-solid ratios and contact times were evaluated. 

## 2. Materials and Methods

### 2.1. Chemicals

Methanol and ethanol of analytical grade (Fisher Chemical, Loughborough, UK) were used as extraction solvents. Folin-Ciocalteu, gallic acid, 6-hydroxy-2,5,7,8-tetramethylchroman-2-carboxylic acid (Trolox), 2,2-diphenyl-1-picrylhydrazyl (DPPH), sodium carbonate, potassium persulfate, 2,4,6-tris(2-pyridyl)-s-triazine (TPTZ), acetic acid, hydrochloric acid, ferric chloride, acetonitrile, and trifluoroacetic acid (TFA) were purchased from Sigma-Aldrich (St. Louise, MO, USA). All the organic solvents were HPLC grade. Ultrapure water from a purified system (Isopad Isomantle, Gemini BV, Pr. Beatrixlaan 301, 7312 DG Apeldoorn, The Netherlands) was used. External standards (purity ≥ 99%) caffeic acid, catechin, chlorogenic acid, diosmetin, ferulic acid, glycitin, isorhamnetin, (-)-epicatechin, luteolin-7-*O*-glucoside, luteolin-4′-*O*-glucoside, myricetin, naringin, p-hydroxybenzoic acid, quercetin, and rutin were purchased from Extrasynthese (Lyon Nord, Genay Cedex, France). Hispidin (purity ≥ 98%) was purchased from Sigma-Aldrich.

### 2.2. Mushroom Material

*I. hispidus* sporocarps were collected in an apple orchard, located in Lamego, North of Portugal, in October 2021. After taxonomic identification at the Laboratory of Mycology of the University of Trás-os-Montes and Alto Douro, the sporocarps were cut into small pieces, dried in a drying oven (Termaks, Nordic Labtech AB, Kungsbacka, Sweden) at 40 °C, and then ground to a fine powder. The samples were kept in the dark in hermetically sealed plastic bags up to analyses.

### 2.3. UAE Methodology

The UAE method was used to extract the bioactive compounds of *I. hispidus*, which is based on the principles of the breakdown of cell walls using ultrasound waves [25,27,29]. Methanol 80% (*v*/*v*) was used as solvent since it is often used for extracting phenolic compounds, partly due to being more economical. Ethanol was also used as a solvent and the choice of a concentration of 40% (*v*/*v*) was based on previous studies carried out in our laboratory, whose extraction of phenolic compounds and antioxidant activity of the extract proved to be better than the other tested concentrations. Firstly, 1 g of dried powder was mixed with 50 mL of 80% (*v*/*v*) methanol or 40% (*v*/*v*) ethanol. The extractions were performed in a pulsed mode of 5 s on/5 s off cycles for 40 min (contact time) using the Hielscher ultrasonic processor device (Hielscher UP400St, Berlin, Germany), with a sonotrode of 14 mm diameter, 400 Watts, 24 kHz, and adjustable amplitude (1:2.55). During the sonication process, the samples were placed in an ice bath to maintain a sample temperature in the range of 40 to 50 °C to avoid thermodegradation of the phenolic compounds [30]. After completion of the extraction, the samples were centrifuged (4500× *g* for 20 min at 4 °C), the supernatants were filtered using a Whatman no. 4 filter paper, collected, and stored at −20 °C until analyses. All the experiments were performed in triplicate. 

### 2.4. Experimental Design

For the previously selected extraction solvent, new experimental assays were carried out using different contact time conditions and solvent-to-solid ratio conditions (Table 1). These variables were combined based on a central composite design. After sonication, each extract was centrifuged and filtered as previously described, and the supernatants were stored until analyses began. The effect of two independent numeric variables, contact time (min, X_1_) and solvent-to-solid ratio (mL/g, X_2_), on the efficiency of the extraction of phenolic compounds, DPPH scavenging capacity, and ferric reducing antioxidant power (FRAP) in *I. hispidus* was evaluated according to a central composite design [31,32]. Variables were coded at the levels −2, −1, 0, 1, and 2 (Table 2), and thirteen runs were established under specific conditions. The content of total phenolics, the radical scavenging capacity (DPPH assay), and FRAP were assessed in the obtained extracts. The model included five central points. To lessen the effect of natural variability on the response, sampling was done randomly. The optimized condition was determined using RSM, while the determination of the significance of primary variable effects, variable interaction, and the model was evaluated using Analysis of Variance (ANOVA). 

### 2.5. Evaluation of TPC

The Folin–Ciocalteu method was used to evaluate the content of phenolic compounds in the extracts as previously described [33], with some modifications. Briefly, 20 μL of each sample extract, 100 μL of Folin-Ciocalteu reagent (10%, *v*/*v*), and 80 μL of aqueous sodium carbonate (7.5%, *w*/*v*) were mixed in a microplate and then incubated at 42 °C during 30 min protected from the light. The absorbance was then measured at 750 nm (Multiskan FC Microplate Photometer, Thermo Fisher Scientific, Vantaa, Finland). The standard curve was obtained using gallic acid (5 to 200 mg/L). Values were expressed as mg of gallic acid per gram of dry weight of raw material (mg GA/g DW).

### 2.6. In Vitro Antioxidant Capacity

The DPPH assay was used to determine the free radical scavenging activity as previously reported [34], with slight modifications. The measurements were performed on a microscale using a 96-well microplate reader (Multiskan FC Microplate Photometer, Thermo Fisher Scientific, Vantaa, Finland). In the DPPH assay, 10 µL of the samples were added to 190 µL of DPPH solution (8.87 mM). The plate was allowed to rest in the dark and the absorbances were read at 520 nm after 15 min of incubation. The antioxidant capacity of the extracts was determined by using the calibration curve with Trolox in a concentration range varying from 0.156 up to 2.500 mM. Values were expressed as millimoles of Trolox per gram of DW of raw material (mmol Trolox/g DW).

The FRAP assay was performed according to Mena et al. [34] with some modifications. A FRAP working solution was prepared by mixing 10-volumes of 300 mM acetate buffer (pH 3.6), 1-volume of 10 mM TPTZ (dissolved in hydrochloric acid), and 1-volume of 20 mM ferric chloride (prepared in distilled water). Samples (20 µL) were placed in a 96-well microplate, and then 280 µL of FRAP working solution (warmed at 37 °C for 10 min) was added. The reaction was incubated at 37 °C for 30 min and the absorbance was read at 593 nm. A calibration curve with Trolox (0.039 to 1.25 mM) was used, and the values were expressed as millimoles of Trolox per gram of DW of raw material (mmol Trolox/g DW).

### 2.7. HPLC Analysis

The profile and content of phenolic compounds from the extract obtained at the optimized condition were analyzed in triplicate by HPLC-DAD, as previously described [35]. Sample extracts (10 μL), in triplicate, were injected into a C18 column (250 × 4.6 mm, 5 μm particle size; ACE HPLC Columns, Advanced Chromatography Technologies Ltd., Abeerden, Scotland, UK) with an eluent composed of water with 0.1% TFA (solvent A) and acetonitrile with 0.1% TFA (solvent B). The elution was performed at a flow rate of solvent of 1 mL/min, with a gradient starting from 0% solvent B at 0 min, 0% solvent B at 5 min, 20% solvent B at 15 min, 50% solvent B at 30 min, 100% solvent B at 45 min, 100% solvent B at 50 min, 0% solvent B at 55 min, and 0% solvent B at 60 min. Chromatograms were recorded in a range of 200–600 nm: 254 and 280 nm for benzoic acids and flavan-3-ols, 320 nm for cinnamic acids, and 370 nm for flavonoids. Phenolics were identified using peak retention time, UV spectra, and UV maximum absorbance band, and by comparison with the literature. Naringin (internal standard) was prepared at a concentration of 2.0 mg/mL in 70% (*v*/*v*) methanol (methanol:water) and run simultaneously with the samples. The amount of each compound was calculated using the internal standard method, and the results were expressed as µg/g DW. 

### 2.8. Statistical Analysis

Firstly, the Mann–Whitney U test (GraphPad Prism 7 Software, Inc.) was used to verify the effect of each solvent on the extraction of polyphenolic compounds with antioxidant capacity from *I. hispidus*. All the assays were carried out in triplicate and the results were expressed as mean ± standard deviation (*n* = 3). Secondly, statistical analysis of the design, to determine the conditions able to maximize the extraction results, was performed with the software Design Expert (version 13.0, Stat-Ease Inc., Minneapolis , MN, USA). The Pearson correlation coefficient for selected pairs of parameters was also estimated. Differences were considered significant when *p <* 0.05.

## 3. Results and Discussion

### 3.1. Determination of the Extraction Solvent

As observed in Table 3, extracts with phenolic compounds and antioxidant capacity were achieved with both ethanol and methanol. These data are consistent with previous works that report ethanol and methanol as the most efficient organic solvents to extract phenolic compounds from different raw materials [28,36,37,38,39,40]. According to our results, ethanol produced extracts with higher and statistically different (*p* < 0.0001) TPC, DPPH, and FRAP values than methanol. In fact, ethanol has been successfully used to extract antioxidant compounds [41], including from mushrooms [42], and due to its lower toxicity compared to methanol, it has been chosen as an organic solvent in different extraction procedures using different raw materials. Accordingly, ethanol was the selected solvent for the subsequent analysis of the study.

### 3.2. Optimization of the Extraction Conditions

**Figure 1:**
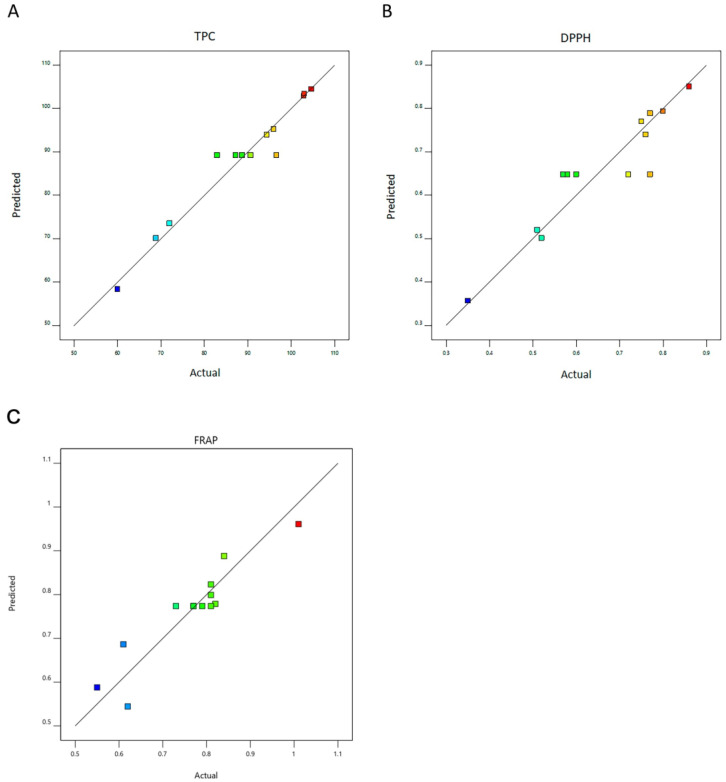
Predicted versus actual values for the RSM design for (**A**) TPC, (**B**) DPPH, and (**C**) FRAP. DPPH, 2,2-diphenyl-1-picrylhydrazyl; FRAP, ferric reducing antioxidant power; RSM, response surface methodology; TPC, total polyphenol content.

Once the extraction solvent has been selected, different extraction conditions were tested to maximize the recovery of antioxidant phenolic compounds from *I. hispidus*. It is well known that various factors affect the antioxidant activity of the extracts and the kinetic of phenolic compounds released from the solid matrix. Those critical variables are directly related to the yield of the extracts and include, for instance, the extraction procedure, the solvent type and concentration, the extraction time, the solvent-to-solid ratio, and the temperature at which the extraction is performed, among others. Using the UAE methodology and based on an experimental design, Ianni et al. [43] described how different variables affect the yield of the extraction process to obtain phenolic compounds from *Pleurotus ostreatus*. In our study, to optimize the extraction conditions to obtain the greatest extraction yields, the contact time employed in the UAE method and the solvent-to-solid ratio used were the variables tested. Table 4 presents the conditions of each experimental assay and the respective measured and predicted values of TPC, DPPH scavenging capacity, and FRAP. The values were analyzed by multiple regression to fit a second-order polynomial equation, and quadratic models describing the variations of the responses as a function of the significant process variables (contact time, X_1_; solvent-to-solid ratio, X_2_—coded values) were established (Table 5). The square coefficient of determination (R^2^) was used to assess the quality of fit, which was 0.95 for total phenolic compounds, 0.87 for DPPH antiradical activity, and 0.86 for FRAP. These findings suggest a highly significant agreement between the results experimentally obtained and those predicted by the equations for TPC, DPPH, and FRAP values, which can adequately predict the experimental results (Figure 1A–C). The model *F* values of 29.00 (TPC), 9.11 (DPPH), and 8.73 (FRAP) and the associated lower *p* value (TPC *p* < 0.001, DPPH *p* < 0.01, and FRAP *p* < 0.01) mean that the generated model is meaningful. 

**Figure 2:**
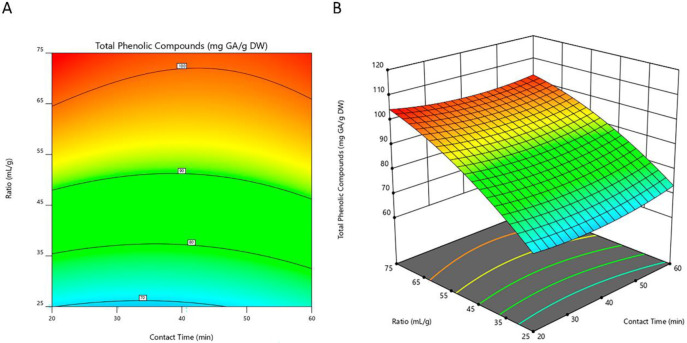
2D-contour line (**A**) and 3D-response (**B**) graphs representing the total content of phenolic compounds of *I. hispidus* extracts obtained by solid-liquid extraction with ethanol at a concentration of 40% (*v*/*v*) under different conditions of contact time and solvent-to-solid ratio. DW, dry weight of raw material; GA, gallic acid.

**Figure 3:**
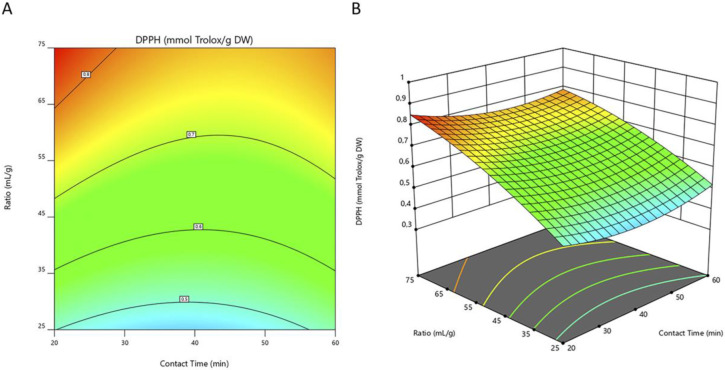
Contour line (**A**) and 3D-response (**B**) graphs representing the antioxidant activity by DPPH assay of *I. hispidus* extracts obtained by solid-liquid extraction with ethanol at a concentration of 40% (*v*/*v*) under different conditions of contact time and solvent-to-solid ratio. DPPH, 2,2-diphenyl-1-picrylhydrazyl; DW, dry weight of raw material.

**Figure 4:**
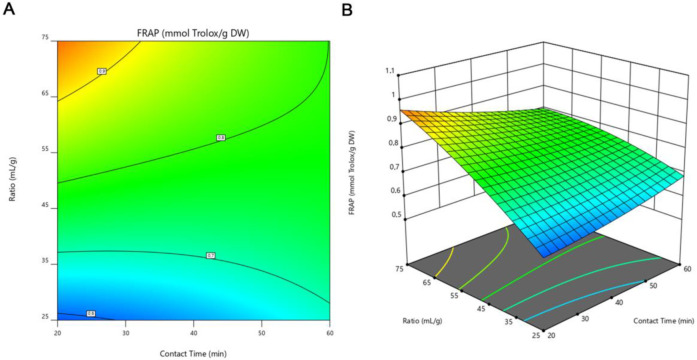
Contour line (**A**) and 3D-response (**B**) graphs representing the antioxidant activity by FRAP assay of *I. hispidus* extracts obtained by solid-liquid extraction with ethanol at a concentration of 40% (*v*/*v*) under different conditions of contact time and solvent-to-solid ratio. FRAP, ferric reducing antioxidant power; DW, dry weight of raw material.

Based on the regression model constructed, a 2D-contour line (Figure 2A, Figure 3A and Figure 4A) and 3D-response graphs (Figure 2B, Figure 3B and Figure 4B) were plotted for each of the responses under analysis. These figures show the similarity between the maximized responses of TCP, DPPH, and FRAP. The results in Table 4 indicate that the highest level of TPC was reached at a ratio of 75 mL/g with a sonication time of 20 min. Likewise, concerning the DPPH and FRAP assays, the highest efficiency was obtained when using those same conditions. As observed, both the content of phenolic compounds as well as the values of antioxidant activity by the DPPH and FRAP assays increased as the solvent-to-solid ratio increased. Likewise, other authors also described, in studies performed with ethanolic extracts from mushrooms [43] and fruits [44], that TPC increased as the solvent-to-solid ratio increased. These results are in accordance with the mass transfer principle where a high solvent-to-solid ratio promotes an increasing concentration gradient. This will further increase the diffusion rate, resulting in a higher extraction of solids by solvent [26,45]. On the other hand, the time of extraction in our study did not significantly affect any of the responses evaluated, which implies that 20 min of sonication could be sufficient for the extraction of the compounds of interest. Ballesteros et al. [31] also aimed to optimize different variables for the ethanolic extraction of antioxidant phenolic compounds from coffee silverskin and found that the time of extraction did not influence either the TPC or the antioxidant activity (evaluated by FRAP and DPPH assays). From an economic perspective, using a shorter extraction time is important as it reduces energy consumption.

**Figure 5:**
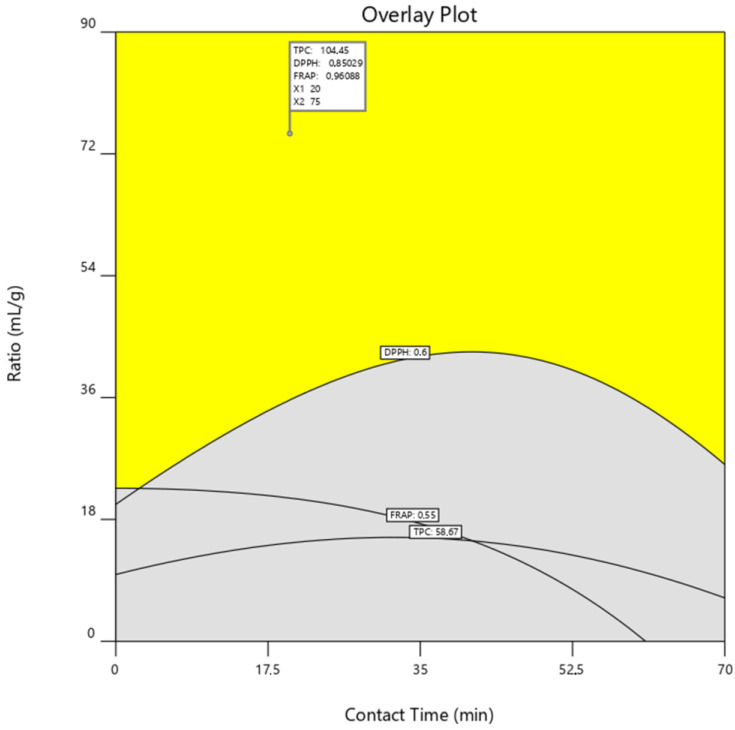
Overlay plot with the optimum point defined for the three evaluated responses as a function of the contact time and solvent-to-solid ratio using UAE methodology. The variables are presented as real values. DPPH, 2,2-diphenyl-1-picrylhydrazyl; FRAP, ferric reducing antioxidant power; TPC, total polyphenol content; UAE, ultrasound-assisted extraction.

In order to confirm the best extraction condition capable of enhancing the responses under analysis, an overlay plot of TPC, DPPH, and FRAP responses was generated. Thus, the following criteria to find the optimal extraction conditions were used: TPC ≥ 58.67 mg GA/g DW, DPPH ≥ 0.6 mmol Trolox/g DW, and FRAP ≥ 0.55 mmol Trolox/g DW. The resulting overlaying plot (Figure 5), obtained by the quadratic polynomial regression model, displayed an area where all the demanded conditions were fulfilled. In this area, an optimum point was chosen corresponding, as expected, to a contact time of 20 min and a ratio of 75 mL/g. The model predicts an extraction of TPC of 104.45 mg GA/g DW, a DPPH value of 0.85 mmol Trolox/g DW, and a FRAP value of 0.96 mmol Trolox/g DW under these conditions.

After determining the best conditions for the two independent variables, extractions were performed in triplicate under those conditions to validate the model. The data obtained for TPC (104.61 ± 13.82 mg GA/g DW), DPPH (0.86 ± 0.15 mmol Trolox/g DW), and FRAP (1.01 ± 0.08 mmol Trolox/g DW) showed a close agreement with the results predicted (TPC, 104.45; DPPH, 0.85; and FRAP, 0.96) by the statistical analysis (Table 6).

### 3.3. Correlation between the Evaluated Responses of I. hispidus Extracts

**Figure 6:**
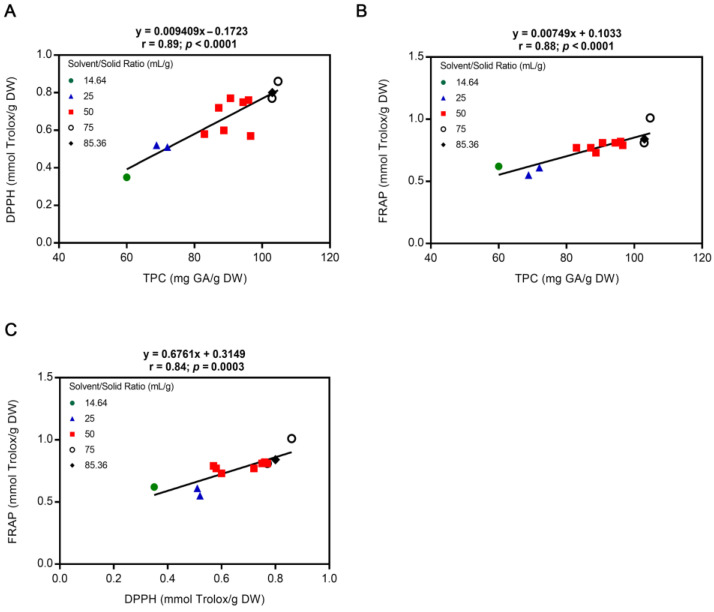
Correlation charts for TPC, DPPH and FRAP responses of the *I. hispidus* extracts using 40% (*v*/*v*) ethanol as solvent. (**A**) TPC vs. DPPH, (**B**) TPC vs. FRAP, and (**C**) DPPH vs. FRAP. At the upper region of the graph, the fitting line equation, Pearson correlation coefficient, and *p* value are shown. DPPH, 2,2-diphenyl-1-picrylhydrazyl; DW, dry weight of raw material; FRAP, ferric reducing antioxidant power; GA, gallic acid; TPC, total polyphenol content.

The correlation between phenolic compounds and the antioxidant activity of extracts from different raw materials has been investigated [46,47]. In the present study, the degree or strength between the results obtained for TPC, DPPH, and FRAP was analyzed by Pearson’s correlation coefficient (*r*). As suggested by Evans [48], the correlation was divided into categories based on the value of the strength to which the two variables are related: very strong (1.0–0.80), strong (0.79–0.60), moderate (0.59–0.40), weak (0.39–0.20), and very weak (0.19–0.00). Correlation analysis charts were plotted (Figure 6) and revealed that DPPH scavenging capacity and FRAP were directly proportional and very strongly correlated with TPC (*r* = 0.89, *p* < 0.0001 and *r* = 0.88, *p* < 0.0001, respectively). Moreover, a statistically significant correlation between DPPH and FRAP values was also observed (*r* = 0.84, *p* < 0.0003). Our results are consistent with other investigations that have demonstrated in mushroom extracts a general correlation between a larger amount of phenolics and a higher antioxidant activity [43,49], which means that phenolic compounds are directly related to the antioxidant potential. However, other studies reported a poor correlation between TPC and DPPH values, which may be due to the presence of other compounds in addition to phenols that could also have DPPH radical scavenging activity [43,50]. Similarly, despite our very strong correlation between DPPH and FRAP values, other authors have reported a weak correlation between them [43,51]. A correlation between DPPH and FRAP means that both assays share a similar mechanism of action, such as electron transfer from antioxidant to oxidant [52].

### 3.4. HPLC Analysis of Phenolic Compounds

The profile and content of phenolic compounds from the extract obtained in optimal condition (20 min and 75 mL/g liquid/solid ratio) was analyzed using HPLC-DAD. The obtained results are in Table 7. Hispidin and hispidin-like compounds (compounds with hispidin-like UV spectrum) accounted for the majority of phenolic compounds in this research (159.56 µg/g out of 219.01 µg/g). Hispidin is a yellow polyphenol pigment known as styrylpyrone, which was isolated and identified for the first time in *I. hispidus* [53]. This compound displays a large number of biological effects, such as anti-cancer, anti-platelet, anti-oxidative, anti-diabetic, anti-inflammatory, and antiviral activities [53,54]. Hispidin has the capacity to neutralize free radicals [17,53,55,56], and a recent study performed in *Phenllinus* mushroom extracts showed that this compound, but not polysaccharides or flavonols, determines its antioxidant and antitumor properties [57]. Gründemann et al. [58] identified hispidin in methanolic extracts of *I. hispidus* as the most prevalent compound. In our investigation, the HPLC profile also demonstrated the presence of other compounds to a lesser extent, such as flavonols (quercetin, myricetin, and isorhamnetin), flavones (luteolin-7-*O*-glucoside, luteolin-4′-*O*-glucoside, and diosmetin), isoflavones (glycitin), phenolic acids (hydroxybenzoic acid), and hydroxycinnamic acids (caffeic acid). According to our results, it is plausible that the antioxidant and reducing power observed in the extract of *I. hispidus* at the optimized condition is mainly due to the presence of hispidin and hispidin-like compounds. 

## 4. Conclusions

In this work, the UAE extraction process of phenolic compounds from *I. hispidus* was optimized by RSM design. Ethanol at 40% (*v*/*v*) was an effective solvent for the extraction of phenolics with antioxidant capacity in the ratio of 75 mL/g DW, during 20 min of sonication. *I. hispidus* is an underexplored mushroom, and for the first time, the best conditions to maximize the extraction of antioxidant phenolic compounds were successfully established. These findings are of great relevance because these compounds have vast functional properties and potential cosmeceutical, pharmaceutical, and food applications. However, although the extract of *I. hispidus* has favorable in vitro potential, the pharmacokinetics and toxicity of the extract compounds must be determined through in vivo toxicological studies.

## Figures and Tables

**Table 1:** Experimental design of the central composite design.

Run Order	Contact Time (min)	Solvent-to-Solid Ratio (mL/g)	Response TPC (mg GA/g DW)	Response DPPH (mmol Trolox/g DW)	Response FRAP (mmol Trolox/g DW)
1	60	25	R1	R1	R1
2	60	75	R2	R2	R2
3	40	50	R3	R3	R3
4	20	25	R4	R4	R4
5	68.28	50	R5	R5	R5
6	40	50	R6	R6	R6
7	40	14.64	R7	R7	R7
8	40	50	R8	R8	R8
9	40	50	R9	R9	R9
10	11.72	50	R10	R10	R10
11	20	75	R11	R11	R11
12	40	50	R12	R12	R12
13	40	85.36	R13	R13	R13

DPPH, 2,2-diphenyl-1-picrylhydrazyl; DW, dry weight of raw material; FRAP, ferric reducing antioxidant power; GA, gallic acid; TPC, total polyphenol content.

**Table 2:** Independent variables and their corresponding levels for hydroethanolic extracts of *I. hispidus*.

Independent Variables	Symbol	Coded Levels				
		−α (−2)	−1	0	+1	+α (+2)
Contact Time (min)	X_1_	11.72	20	40	60	68.28
Solvent-to-Solid Ratio (mL/g)	X_2_	14.64	25	50	75	85.36

**Table 3:** TPC and antioxidant capacity (DPPH and FRAP assays) of *I. hispidus* extracts obtained using different organic solvents.

Contact Time	Organic Solvent	TPC (mg GA/g DW)	DPPH (mmol Trolox/g DW)	FRAP (mmol Trolox/g DW)
40 min	Ethanol 40% (*v*/*v*)	100.70 ± 5.08 ****	1.09 ± 0.08 ****	0.78 ± 0.03 ****
	Methanol 80% (*v*/*v*)	85.94 ± 2.41	0.88 ± 0.05	0.69 ± 0.03

Data presented as mean ± SD of three replicates. The means of both solvents were compared by Mann–Whitney test, **** *p* < 0.0001. DPPH, 2,2-diphenyl-1-picrylhydrazyl; DW, dry weight of raw material; FRAP, ferric reducing antioxidant power; GA, gallic acid; TPC, total polyphenol content.

**Table 4:** Effect of processing variables on TPC, DPPH scavenging activity, and FRAP of hydroethanolic extracts of *I. hispidus* by RSM.

Assay	Coded Level (Real Values)		TPC (mg GA/g DW)		DPPH (mmol Trolox/g DW)		FRAP (mmol Trolox/g DW)	
	Contact Time (min)	Solvent-to-Solid Ratio (mL/g)	Observed	Predicted	Observed	Predicted	Observed	Predicted
1	+1 (60)	−1 (25)	72.00	73.47	0.51	0.52	0.61	0.69
2	+1 (60)	+1 (75)	102.89	102.87	0.77	0.79	0.81	0.80
3 ^Z^	0 (40)	0 (50)	96.60	89.22	0.57	0.65	0.79	0.77
4	−1 (20)	−1 (25)	68.83	70.08	0.52	0.50	0.55	0.59
5	+2 (68.28)	0 (50)	95.96	95.19	0.76	0.74	0.82	0.78
6 ^Z^	0 (40)	0 (50)	82.94	89.22	0.58	0.65	0.77	0.77
7	0 (40)	−2 (14.64)	60.00	58.33	0.35	0.36	0.62	0.54
8 ^Z^	0 (40)	0 (50)	88.71	89.22	0.60	0.65	0.73	0.77
9 ^Z^	0 (40)	0 (50)	90.66	89.22	0.77	0.65	0.81	0.77
10	−2 (11.72)	0 (50)	94.38	93.91	0.75	0.77	0.81	0.82
*11*	*−1 (20)*	*+1 (75)*	*104.68*	*104.45*	*0.86*	*0.85*	*1.01*	*0.96*
12 ^Z^	0 (40)	0 (50)	87.19	89.22	0.72	0.65	0.77	0.77
13	0 (40)	+2 (85.36)	103.00	103.43	0.80	0.79	0.81	0.89

^Z^ Central point. In italics, the best condition for each of the tested variables was highlighted. DPPH, 2,2-diphenyl-1-picrylhydrazyl; DW, dry weight of raw material; FRAP, ferric reducing antioxidant power; GA, gallic acid; RSM, response surface methodology; TPC, total polyphenol content.

**Table 5:** *F* values and *p* values for each coefficient and polynomial equations calculated by the central composite design for the extraction conditions of *I. hispidus*.

Responses	Statistics	X_1_	X_2_	X_1,2_	X_1_^2^	X_2_^2^	Model
TPC	*p* value	0.7661 (n.s.)	<0.0001 (****)	0.5477 (n.s.)	0.1164 (n.s.)	0.0265 (*)	0.0002 (***)
	*F* value	0.1060	131.94	0.3990	3.21	7.84	29.00
DPPH	*p* value	0.6801 (n.s.)	0.0004 (***)	0.5886 (n.s.)	0.0857 (n.s)	0.2148 (n.s.)	0.0057 (**)
	*F* value	0.1850	38.35	0.3212	4.00	1.86	9.11
FRAP	*p* value	0.4650 (n.s.)	0.0006 (***)	0.0586 (n.s.)	0.5524 (n.s.)	0.2276 (n.s.)	0.0064 (**)
	*F* value	0.5970	35.54	5.10	0.3894	1.75	8.73
TPC = 89.22 + 0.45X_1_ + 15.9439X_2_ − 1.24X_1_X_2_ + 2.66625X_1_^2^ − 4.16875X_2_^2^; R^2^ = 0.95							
DPPH = 0.648 − 0.0107322X_1_ + 0.15455X_2_ − 0.02X_1_X_2_ + 0.0535X_1_^2^ − 0.0365X_2_^2^; R^2^ = 0.87							
FRAP = 0.774 − 0.0157322X_1_ + 0.121391X_2_ − 0.065X_1_X_2_ + 0.013625X_1_^2^ − 0.028875X_2_^2^; R^2^ = 0.86							

DPPH, 2,2-diphenyl-1-picrylhydrazyl; FRAP, ferric reducing antioxidant power; TPC, total polyphenol content; X_1_, contact time (min); X_2_, solvent-to-solid ratio (mL/g). n.s., not significant; * *p* < 0.05, ** *p* < 0.01, *** *p* < 0.001, **** *p* < 0.0001.

**Table 6:** Validation of the optimal extraction conditions for TPC, DPPH, and FRAP from *I*. *hispidus*.

Experimental Assays	Independent Variables		Responses		
	Contact Time (min)	Solvent-to-Solid Ratio (mL/g)	TPC (mg GA/g DW)	DPPH (mmol Trolox/g DW)	FRAP (mmol Trolox/g DW)
1	20	75	121.27 ± 2.11	1.01 ± 0.14	1.11 ± 0.02
2	20	75	95.72 ± 1.14	0.77 ± 0.02	0.98 ± 0.01
3	20	75	96.84 ± 1.62	0.80 ± 0.19	0.93 ± 0.01
Average			104.61 ± 13.82	0.86 ± 0.15	1.01 ± 0.08
Results predicted by the statistical analysis			104.45	0.85	0.96

Data presented as mean ± SD of three replicates. DPPH, 2,2-diphenyl-1-picrylhydrazyl; DW, dry weight of raw material; FRAP, ferric reducing antioxidant power; GA, gallic acid; TPC, total polyphenol content.

**Table 7:** Phenolic compounds identified and quantified from *I. hispidus* extracts at the optimized condition.

Compound	Retention Time (min)	Concentration (µg/g DW)
Glycitin	5.62	2.26 ± 0.006
Diosmetin	8.03	18.39 ± 0.050
Hydroxybenzoic acid	12.32	3.48 ± 0.032
Caffeic acid	19.42	1.24 ± 0.012
Luteolin-7-*O*-glucoside	20.64	1.22 ± 0.033
Myricetin	20.91	13.45 ± 0.130
Luteolin-4′-*O*-glucoside	22.27	3.75 ± 0.087
Quercetin	22.37	1.91 ± 0.030
Hispidin	23.61	122.80 ± 1.456
Hispidin-like compound	24.98	4.08 ± 0.115
Hispidin-like compound	26.37	3.86 ± 0.095
Hispidin-like compound	27.31	5.70 ± 0.057
Hispidin-like compound	29.11	19.50 ± 0.101
Hispidin-like compound	30.11	3.62 ± 0.101
Isorhamnetin	33.90	13.75 ± 0.035

Data presented as mean ± SD of three replicates. DW, dry weight of raw material.

## Data Availability

Dataset will be provided upon reasonable request. Data is not publicly available due to privacy.
